# Noncoding RNAs in nuclear organization

**DOI:** 10.1080/19491034.2025.2477848

**Published:** 2025-03-13

**Authors:** Thembalami Dube, Dawn M. Carone

**Affiliations:** Department of Biology, Swarthmore College, USA

RNA has long been recognized as a structural element of the nucleus. Biochemical studies in the 1960s established a ribonucleoprotein ‘matrix’ [1] and have revealed that when RNA is removed or transcription is inhibited, this leads to massive chromatin collapse [2]. This RNA component had been hypothesized to consist of noncoding RNAs (ncRNAs) [3], and more recent findings suggest that many nuclear ncRNAs exist within dynamic ribonucleoprotein complexes. Nuclear ncRNAs act as structural platforms for nuclear architecture, play key roles in genome regulation and RNA processing, modulate the immune response and stress responses, and regulate key cellular processes such as translation and DNA damage repair. Thus, proper regulation of nuclear ncRNAs is essential for cellular function, and misregulation of ncRNAs can lead to disease and cell death. The articles in this collection establish and highlight some of the regulatory roles for ncRNA within the nucleus as well as the implications of their misregulation in disease. Emerging properties of ncRNAs, including their ability to form and exist within nuclear condensates, have enhanced our understanding of their myriad roles and have unlocked novel strategies for the development of potential therapeutic targets.

Long non-coding RNAs (lncRNAs) are increasingly recognized as key mediators of genome stability, particularly in how cells adapt to damage, environmental fluctuations, and transcriptional stress. In their comprehensive synthesis, Nickerson and Momen-Heravi [4] present a nuanced exploration of how these RNAs act as molecular sentinels – recruiting chromatin remodelers, buffering transcriptional noise, and even reinforcing nuclear compartments under duress. The review challenges conventional boundaries between transcription and genome organization, showing that these RNAs do not merely respond to cellular signals but also actively sculpt chromatin landscapes. It builds on emerging evidence that lncRNAs engage in a reciprocal relationship with nuclear structure, where transcriptional output is not just a consequence but a regulator of genome topology. Particularly striking is the discussion of lncRNAs in damage control – how specific transcripts tether chromatin loops, stabilize epigenetic marks, and even dictate the formation of nuclear condensates that preserve transcriptional fidelity. By weaving together recent genome-wide studies with mechanistic insights, the authors craft a narrative that positions lncRNAs as indispensable architects of nuclear resilience.

The nucleolus is a unique membraneless subcompartment of the nucleus that is relatively devoid of DNA but instead consists primarily of RNA and ribonucleoprotein complexes. Study of the nucleolus has long focused on the role of ncRNA, since ribosomal RNA constitutes 80% of bulk cellular RNA, and snoRNAs regulate rRNA processing [5]. Yet also within the nucleolus are many ncRNAs that are not directly involved in ribosome synthesis. Böğürcü-Seidel [6] summarizes the role of lncRNAs and miRNAs within the nucleolus, highlighting their roles in gene regulation, chromatin remodeling, and cell cycle regulation. Further, changes in the expression of ncRNAs within the nucleolus can be triggered by cellular stress and lead to cancer development. This review highlights the methods that have been developed to identify nucleolar RNAs, which have led to the discovery of nearly all categories of ncRNA, and summarizes the key lncRNAs and miRNAs with a focus on those that are known to be dysregulated in cancer. The authors highlight the integral role that nucleolar ncRNAs play in nuclear architecture, including RNA and protein sequestration and heterochromatin formation.

The immune response is a highly coordinated process that extends beyond protein-mediated signaling to the realm of nuclear organization, where long non-coding RNAs (lncRNAs) have emerged as key regulators of immune gene expression. Montano et al. [7] unravel the intricate interplay between lncRNAs, chromatin accessibility, and immune activation, highlighting the architectural role of lncRNAs in shaping the nuclear environment during inflammatory responses. This work underscores how nuclear-localized lncRNAs, such as *NEAT1* and *UMLILO*, serve as molecular scaffolds that regulate immune gene priming and transcriptional memory. *NEAT1*, central to paraspeckle formation, modulates antiviral cytokine production, while *UMLILO* directs chromatin looping to epigenetically prime inflammatory chemokines. Beyond structural roles, the study also provides mechanistic insights into how lncRNAs fine-tune immune gene expression by orchestrating transcription factor recruitment and chromatin remodeling. By positioning lncRNAs as both architects and regulators of immune responses, this comprehensive review challenges traditional paradigms of immune regulation, demonstrating that these RNAs do not merely respond to inflammation but actively rewire nuclear architecture to control it.

The discovery that RNA-driven phase separation governs essential nuclear processes has opened new avenues in understanding genome regulation, and Wang et al. [8] unveils an unexpected player in this paradigm. They establish ciRS-7, a circular RNA, as a key regulator of liquid–liquid phase separation (LLPS) within the miRNA-induced silencing complex (miRISC), demonstrating its capacity to orchestrate RNA-protein condensates and enhance DNA repair. By leveraging a suite of *in vivo* and *in vitro* approaches – including fluorescence recovery after photobleaching (FRAP), high-resolution live-cell imaging, and precise biochemical reconstitution – the authors provide striking evidence that ciRS-7 increases AGO2-TNRC6B condensates, thereby amplifying miRISC activity in post-transcriptional gene silencing. Crucially, this work uncovers an unanticipated link between RNA-guided LLPS and homologous recombination, showing that ciRS-7-driven condensates facilitate AGO2-mediated recruitment of RAD51 to DNA damage sites. These findings extend the emerging principle that non-coding RNAs, far from being passive scaffolds, actively modulate nuclear biochemistry through multivalent interactions. By placing a circular RNA at the center of genome maintenance, this study redefines the functional landscape of non-coding RNAs, illuminating how RNA-driven condensates coordinate gene regulation with genome stability – a connection with profound implications for both fundamental biology and therapeutic innovation.

Long dismissed as static cytological markers, cytogenetic bands emerge as dynamic regulators of nuclear architecture in this recent study from the Lawrence lab. Hall et al. [9] reveal that these large-scale genomic compartments shape chromatin behavior through the differential properties of L1- and Alu-rich regions, providing a new lens through which to view genome organization. By integrating Hi-C, DNA FISH, and cellular senescence models, they demonstrate how finely tuned experimental designs can reveal fundamental principles of genome regulation. Their findings show that L1-rich domains, long associated with transcriptional repression, actively coalesce into heterochromatin, while Alu-rich, gene-dense regions resist compaction. This fundamental partitioning is not random, but appears to be exploited by lncRNAs, such as XIST, which selectively leverages L1-dense regions to drive chromosome-wide silencing. Alu elements, in contrast, associate with transcriptionally permissive nuclear hubs, reinforcing their role as architectural boundary elements. These findings force a reevaluation of repetitive elements – not as genomic relics but as intrinsic scaffolds of chromatin organization, bridging genome sequences with lncRNA-mediated nuclear compartmentalization.

Repetitive sequences reemerge as a theme in Rabeler et al. [10], where the expressed sequence variants of Human Satellite II (HSATII) and Human Satellite III are identified in cancer cells and heat shocked cells, respectively. HSat2 RNA has previously been shown to accumulate and recruit proteins with intrinsically disordered domains, such as MeCP2, in CAST bodies within cancer cells. Similarly, HSat3 was known to be induced upon heat shock, resulting in HSat3 RNA accumulation in large nuclear condensates called stress bodies [11]. While the presence of HSAT2 CAST bodies in cancer cells had been previously observed [12], the expressed HSat2 sequence variants remained to be characterized and mapped. Harnessing the CenSat annotations from the recently completed human T2T genome [13], Rabeler et al. developed a computational pipeline for differential expression analysis of nuclear-restricted, tandemly repeated human satellite sequences and identified locus-specific expression of HSat2 sequence variants in cancer cell lines. This study, from our lab, reveals specific subfamilies from distinct genomic loci of HSat2 that are overexpressed and accumulate in the nuclei of cancer cells. The identification of these transcripts can be harnessed to identify novel interacting partners and develop potential therapeutics targeting these expressed sequence variants in cancer cells.

Taken together, these articles highlight the dynamic and emerging roles of ncRNA in genome regulation, stress and immune response, DNA damage response, cancer, and nuclear organization. It is clear that ncRNAs of all types have important structural and regulatory roles within the nucleus and the identification and study of these RNAs have revealed key insights into our understanding of nuclear organization and gene regulation. With the advent of improved sequencing technology and fully completed genome assemblies, the characterization of ncRNAs within the nuclear environment remains ongoing. As the study of nuclear ncRNAs progresses from identification to mechanisms governing their regulation and innumerable functions, some key principles are beginning to emerge. While each unique ncRNA has its own function within the nuclear milieu, studies aimed at the shared properties of these RNAs, including their structural properties (e.g. RNA folding principles and inter- and intra-molecular interactions), the dynamics of their interactions with other nuclear molecules, and their abilities to participate in and/or initiate liquid–liquid phase separation into condensates within the nuclear environment are likely to reveal some shared mechanisms of action. The field of study of ncRNAs within the nuclear environment is still young, emerging and gaining momentum. We thank these authors for their contributions to this Collection, and for allowing us to highlight some of the key contributions their labs are making to this exciting area of RNA biology.
